# Development of a New Purity Certified Reference Material of Gamma Linolenic Acid Methyl Ester

**DOI:** 10.1002/fsn3.70354

**Published:** 2025-06-05

**Authors:** Weizhu Chen, Wenhui Jin, Hua Fang, Hui Chen, Xiaoyuan Huang, Yanrou Jie, Zhuan Hong, Yiping Zhang

**Affiliations:** ^1^ Engineering Research Center of Marine Biological Resource Comprehensive Utilization Third Institute of Oceanography, Ministry of Natural Resource Xiamen Fujian P. R. China; ^2^ Xiamen Ocean Vocational College Xiamen Fujian P. R. China; ^3^ Fujian Provincial Key Laboratory of Island Conservation and Development (Island Research Center, MNR) Pingtan Fujian P. R. China; ^4^ Zhejiang Wanli University Ningbo Zhejiang P. R. China

**Keywords:** certified reference material, characterization, evening primrose oil, gamma linolenic acid methyl ester, homogeneity test, stability study

## Abstract

In the study, a new purity certified reference material (CRM) of gamma‐linolenic acid methyl ester (GLA‐ME), designated as (GBW (E) 091214), was first developed in accordance with ISO Guides 17034 and 35. The research involved comprehensive investigations into the preparation, structure determination, characterization, homogeneity testing, stability testing, and uncertainty evaluation of the GLA‐ME purity CRM. It was prepared by the preparative high‐performance liquid chromatography (Pre HPLC) and molecular distillation (MD) method using evening primrose oil (EPO) as a raw material. The identity of GLA‐ME was confirmed using high‐resolution mass spectrometry (HRMS), nuclear magnetic resonance (NMR), and infrared spectrophotometry (IR). The purity of GLA‐ME was accurately determined by the mass balance (MB) and further validated via quantitative nuclear magnetic resonance (*q*NMR). The homogeneity, long‐term stability, short‐term stability, and uncertainty were systematically studied using the MB. The uncertainty was evaluated by combining the contributions from characterization, homogeneity, and stability. The certified value was 98.9% ± 0.4% (*k* = 2) with 12 months stability under −18°C condition and 5 days stability at room temperature. The CRM was sufficiently homogeneous between and within bottles. It can be used for quality control and method validation to ensure the accuracy and reliability of GLA measurements for quality monitoring in the food and pharmaceutical industries.

## Graphical abstract

In this study, a new purity certified reference material (CRM) of gamma‐linolenic acid methyl ester (GLA‐ME) (GBW (E) 091214) was first developed. The preparation, structure determination, characterization, homogeneity test, stability test, and uncertainty evaluation of the GLA‐ME purity CRM have been investigated. The mass balance (MB) and quantitative proton nuclear magnetic resonance (qNMR) methods were used for characterizing the CRM. The certified value was 98.9% ± 0.4%, with a coverage coefficient *k* value of 2.

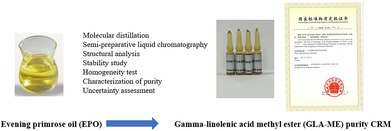

## 1 Introduction

A certified reference material (CRM) is defined as a reference material characterized by using a metrologically valid procedure for one or more specified attributes, accompanied by a certificate that provides the value of the specified property, its associated uncertainty, and a statement of metrological traceability (Wise 2018). This CRM is essential for validating analytical methods, maintaining accuracy, and supporting laboratory quality control in the food and pharmaceutical industries.

Gamma linolenic acid (GLA), also known as cis‐6, cis‐9, cis‐12‐octadecatrienoic acid, is an 18‐carbon polyunsaturated fatty acid (PUFA) with three double bonds that is a member of ω‐6 PUFAs (Cui et al. 2018). It is mainly present in fish and plants such as soybean, grapes, and sunflowers. It is a minor component of animal and human fats and an intermediate in the biosynthesis of biologically active hormones (prostaglandins, thromboxanes, and leukotrienes) (Li et al. 2018). GLA has garnered significant scientific interest over the past four decades due to its diverse physiological functions, including anti‐inflammatory and anti‐cancer properties. Additionally, GLA enhances nerve conduction velocity in diabetic patients, particularly those with type II diabetes, resulting in improved blood circulation and reduced tingling sensations in the extremities (Vasilakis et al. 2025; Prado and Adiao 2024). Many health and dietary studies claim that GLA prevents or alleviates a variety of human and animal diseases. It is an essential fatty acid that must be present in the daily diet for a healthy lifestyle. As a crucial dietary component for maintaining optimal health, GLA must be obtained through daily nutrition since endogenous production in vivo is physiologically limited. This has spurred significant scientific and industrial interest in the exploration, development, and commercialization of sustainable and efficient sources of GLA (Vasilakis et al. 2025). Now, many sources of GLA have been commercialized. Commercial quantities of GLA are produced from the seed oils of borage ( *Borago officinalis* ) (20%–25% GLA), blackcurrant ( *Ribes nigrum* ) (15%–17% GLA), and evening primrose ( *Oenothera biennis* ) (7%–14% GLA) (Lyashenko et al. 2021; Shahinfar et al. 2023; Kim et al. 2024). Different sources have different amounts of GLA and different prices. In some cases, the amount of GLA in some sources is not consistent with what is indicated on their labels. Therefore, it is important that the content of GLA in different sources ought to be accurately measured. GLA is usually determined by the GC‐FID method based on its methyl ester form that is gamma‐linolenic acid methyl ester (GLA‐ME) (Li et al. 2018; Suryawanshi and Mohanty 2018). To our knowledge, no such CRM has been developed now. When researchers in different laboratories make the same measurements, the results are difficult to compare and to distinguish between their quality and reliability. Therefore, GLA‐ME purity CRM was developed in this paper.

GLA‐ME purity CRM (GBW (E) 091214) was prepared according to International Standard Organization (ISO) guide and Chinese National Technical Specifications of Metrology JJF 1855‐2020 in the study (ISO 2016,2017; Station Administration for Market Regulation 2021). The preparation, structure analysis, homogeneity and stability evaluation, certified value determination, and uncertainties estimation for the CRM were studied. The high‐purity GLA‐ME candidate CRM was prepared by the Pre HPLC and MD using EPO as a raw material. The structural characterization of the GLA‐ME purity CRM was comprehensively established through spectroscopic analysis, including IR for functional group identification, HRMS for molecular weight determination, and both ^1^H NMR and ^13^C NMR for detailed molecular structure elucidation. The defined value was calculated by the mean obtained by mass balance (MB) and *q*NMR. The certification procedure in the MB comprised determination of the main component by GC‐FID, of nonvolatile impurities by ICP‐MS, and of volatile impurities by headspace GC‐FID, and of moisture by Karl‐Fischer titration.

## 2 Materials and Methods

### 2.1 Chemicals and Materials

EPO was bought from Yuanye Co. Ltd. (Shanghai, China). Methyl sulfoxide‐*d*_*6*_ (DMSO‐*d*_*6*_) (> 99.9%, 0.03%, v/v, TMS) was purchased from Qingdao tenglong Co. Ltd. (Qingdao, Shandong, China). Water Content of Solid (GBW 13518, (9.90 ± 0.20) mg∙g^−1^), Water Content of Lactose Monohydrate CRM (GBW 13517, (50.07 ± 0.6) mg∙g^−1^), Water Content of Sodium Tartrate Dihydrate (GBW 13515, (156.3 ± 1.3) mg∙g^−1^), and the benzoic acid CRM (GBW 06117, (99.990 ± 0.009) %) were from the National Institute of Metrology, China. HPLC grade methanol and hexane were from Sigma Aldrich (St. Louis, MO, USA). Ultrapure water was prepared by a Milli‐Q system at 18.2 MΩ.cm (Millipore, Bedford, MA, USA).

The GC system consisted of an Agilent 7890B chromatograph (Agilent, Waltham, MA, USA) equipped with a flame ionization detector and a headspace injector. Distillation of the GLA‐ME was carried out in a molecular distiller (KDL2‐UIC Short Path Distillation, Germany) that consisted of a feed tank connected to a frequency inverter and a short path evaporator. The preparative separations were obtained with a Pre HPLC system (Habon Sci. &Tech.) equipped with an NP 7000 serial pump and a NU 3000 serial UV/vis Detector. The ^1^H NMR and ^13^C NMR of the compounds were measured on a Bruker AV400 spectrometer (Bruker, Karlsruhe, Baden‐Württemberg, Germany). Waters Xevo G2 QTof (Waters, Milford, MA, USA) and Bruker Tensor 27 (Bruker, Karlsruhe, Baden‐Württemberg, Germany) were used to perform HRMS and IR for the compounds, respectively. A Karl–Fischer titrator (810 model) (Metrohm AG, Bleiche West, Switzerland) was used for determining the moisture measurement of the sample. The nonvolatile impurities of GLA‐ME candidate CRM were analyzed by the iCAPQ ICP‐MS (Thermo Fisher Scientific, Waltham, MA, USA). Sartorius SOP and Mettler ME104 analytical balances were used to weigh the sample.

### 2.2 Methods

#### 2.2.1 Preparation of the Candidate CRM

Initially, 300 g of EPO was charged into a 2 L round‐bottom flask, followed by the sequential addition of 750 g of methanol and 0.75 g of sodium hydroxide. The reaction mixture was maintained at 60°C with continuous stirring for 16 h to ensure complete transesterification. The resulting mixture was then subjected to primary MD using a KDL2‐UIC system under optimized parameters: evaporation temperature of 95°C, rotational speed of 175 rpm, inner condenser temperature of 40°C, feed rate of 6 mL min^−1^, and vacuum pressure of 1.0 × 10^−3^ mbar. The collected light fraction from the primary distillation was further purified through secondary MD under enhanced conditions: evaporation temperature of 150°C, rotational speed of 220 rpm, inner condenser temperature of 40°C, feed rate of 20 mL min^−1^, and vacuum pressure of 1.0 × 10^−3^ mbar. Finally, the secondary MD product was further refined using preparative Pre‐HPLC with the following operational parameters: ZORBAX‐SB‐C18 column (21.2 × 150 mm, 5 μm), mobile phase consisting of methanol/water (9:1, v/v) at a flow rate of 15.0 mL min^−1^, detection wavelength of 205 nm, and injection volume of 500 μL.

Following purification, the purified GLA‐ME was removed from organic solvents through evaporation under reduced pressure and then sealed under N_2_ protection in 200 brown ampoules. Each ampoule was filled with approximately 50 mg of purified GLA‐ME and subsequently flame‐sealed to ensure complete isolation from atmospheric oxygen and moisture. All the sealed ampoule bottles were stored at −18°C as the GLA‐ME candidate CRM.

#### 2.2.2 Structural Analysis

##### 2.2.2.1 High‐Resolution Mass Spectra

HRMS analysis of the GLA‐ME candidate CRM was conducted using electrospray ionization (ESI) operated in positive ion mode, with mass spectra acquired across the *m/z* range of 100–1000. The ionization source parameters were optimized as follows: source temperature maintained at 100°C, desolvation temperature set at 350°C, cone gas (N_2_) flow rate of 10 L∙h^−1^, desolvation gas (N_2_) flow rate of 40 L∙h^−1^, capillary voltage of 3.0 kV, and sample cone voltage of 50 V.

##### 2.2.2.2 Fourier‐Transform Infrared (FT‐IR) Spectra

IR spectra of the GLA‐ME candidate CRM was acquired via the attenuated total reflection (ATR) technique. Spectra were collected in the mid‐infrared region ranging from 4000 to 650 cm^−1^ with a spectral resolution of 4 cm^−1^. Each measurement represented the average of 32 consecutive scans to ensure an optimal signal‐to‐noise (S/N) ratio, and background spectra were collected under identical conditions prior to sample analysis to enable proper spectral correction.

##### 2.2.2.3 Nuclear Magnetic Resonance Spectra

For nuclear magnetic resonance (NMR) spectroscopic analysis, a precisely weighed quantity of the GLA‐ME candidate CRM was dissolved in DMSO‐*d*_*6*_ to prepare a homogeneous solution with a concentration of 10.0 ± 0.2 mg∙mL^−1^. Both ^1^H and ^13^C NMR spectra were acquired at 298 K using a high‐field NMR spectrometer operating at 500 MHz. The acquired spectra were processed and analyzed using advanced NMR software, with structural assignments verified against the established molecular structure of GLA‐ME. Chemical shifts were referenced to the residual solvent peak (DMSO‐*d*_6_) at 2.50 ppm for ^1^H NMR and 39.52 ppm for ^13^C NMR, respectively.

### 2.3 Characterization of Purity

#### 2.3.1 Mass Balance

The MB approach was systematically implemented to determine the absolute purity of the GLA‐ME candidate CRM. This comprehensive methodology involves the precise quantification of both the main component and all detectable impurities, including moisture content, volatile organic compounds, and nonvolatile residues. The fundamental principle of MB dictates that the sum of these quantified components must equal 100% of the sample mass. The purity assessment was conducted through a two‐step analytical process: first, the cumulative percentage of all identified impurities was determined through validated analytical methods; second, this total impurity content was subtracted from 100% to yield the absolute purity value. The purity of the GLA‐ME candidate CRM was calculated using the following Equation (1): (Chen, Jin, Fang, et al. 2022; Chen, Jin, Zhang, et al. 2022).

(1) $$P_{\mathrm{MB}}=P_{0}\;\left(1-X_{V}-X_{W}-X_{\mathrm{NV}}\right)$$

where $P_{\mathrm{MB}}$,$P_{0}$, $X_{V}$, $X_{W},$ and $X_{\mathrm{NV}}$ refer to the purity of the GLA‐ME candidate CRM, main component, volatile impurities, moisture, and nonvolatile impurities, respectively.

#### 2.3.2 Structurally Related Impurities by GC‐FID

The quantitative analysis of the main component and structurally related impurities in the GLA‐ME candidate CRM was performed using gas chromatography with GC‐FID. An accurately weighed quantity of the candidate CRM was dissolved in hexane to prepare a stock solution with a concentration of 2.00 mg∙mL^−1^. Chromatographic separation was achieved using a DB‐FFAP capillary column (30 m × 0.25 mm × 0.25 μm) with the following optimized operating conditions: injection port temperature maintained at 260°C, detector temperature set at 270°C, and oven temperature program initiated at 100°C (hold for 5 min), followed by a linear temperature gradient of 5°C∙min^−1^ to 220°C (maintained for 24 min). The carrier gas (H_2_) flow rate was maintained at 1.0 mL∙min^−1^, with auxiliary nitrogen flow rates of 30 mL∙min^−1^ and combustion nitrogen at 40 mL∙min^−1^. The combustion‐supporting air flow was set at 400 mL∙min^−1^. Sample introduction was performed using a 1.0 μL injection volume with a split ratio of 10:1. For method validation and calibration, a series of working standard solutions were freshly prepared at concentrations of 0.10, 0.20, 0.60, 1.00, 2.00, 4.00, and 6.00 mg∙mL^−1^ by serial dilution of the stock solution. These calibration standards were analyzed under identical chromatographic conditions to establish the quantitative relationship between peak area and analyte concentration.

#### 2.3.3 Determination of Other Impurities

The moisture, volatile impurities, and nonvolatile impurities of the GLA‐ME candidate CRM were determined by Karl‐Fischer titration, head‐space GC–FID, and ICP–MS, respectively (ISO 2017).

#### 2.3.4 qNMR Method

*q*NMR is employed based on the fundamental principle that the signal response (integral signal area I_X_) is proportional to the number of nuclei producing the corresponding resonance line. In this study, CRM of benzoic acid (GBW 06117) was utilized as an internal standard (IS). A representative ^1^H NMR spectrum was acquired for the sample solution containing both the IS and GLA‐ME candidate CRM under optimized experimental conditions. The purity of the GLA‐ME candidate CRM was determined as follows (Chen, Jin, Fang, et al. 2022; Chen, Jin, Zhang, et al. 2022):

(2) $$P_{x}=I_{x}/I_{\mathrm{std}}\cdot N_{\mathrm{std}}/N_{x}\cdot M_{x}/M_{\mathrm{std}}\cdot m_{\mathrm{std}}/m_{x}\cdot P_{\mathrm{std}}$$

where P, I, N, M, and m represent the purity of the sample, the areas of the integrated signal, the spin numbers the molar mass and the mass, respectively; subscript x and std represent the GLA‐ME candidate CRM and IS.

For *q*NMR analysis, precisely weighed quantities of the GLA‐ME candidate CRM and IS were dissolved in DMSO‐*d*_6_ and volumetrically diluted to 5 mL to prepare solutions across a concentration range for method optimization. ^1^H NMR spectra were acquired under the following optimized parameters: probe temperature maintained at 25.0°C, 5 mm broadband probe, 45° excitation pulse angle, 16 K spectral data points, 32 K time‐domain points, 30 s relaxation delay, 4.15 μs pulse delay, and 32 accumulated scans. The certified concentration was determined through comparing the integration results of samples with different concentrations. Initial concentration determination was performed through comparative integration analysis of solutions at varying concentrations, establishing 1.00 mg mL^−1^ as the optimal certified concentration for both the IS and GLA‐ME candidate CRM. For final quantification, six replicate samples were prepared by accurately weighing approximately 5.00 mg of both the GLA‐ME candidate CRM and IS, followed by dilution to a final volume of 5 mL with DMSO‐*d*_6_. The six samples were analyzed and the purity of the GLA‐ME candidate CRM was formulated by Equation (2).

### 2.4 Homogeneity Test

For homogeneity assessment, 15 bottles of the GLA‐ME candidate CRM were selected from the entire batch using a statistically designed sampling plan, and three subsamples from each bottle were analyzed by GC‐FID in triplicate. The data was analyzed using variance (ANOVA, *F*‐test) (Kim et al. 2024; Suryawanshi and Mohanty 2018). The criterion of the *F*‐test is shown below:

(3) $$F=\frac{{\mathrm{MS}}_{\text{among}}}{{\mathrm{MS}}_{\text{within}}}<F\left(n,m\right)$$

where *n* and *m* represent the numbers of detections for among bottles and within bottle, respectively. $F_{\alpha }\left(n,m\right)$ can be seen from the *F*‐test list.

### 2.5 Stability Study

The stability of the GLA‐ME candidate CRM was evaluated through both short‐term and long‐term studies. For the short‐term stability assessment, 15 bottles of the candidate CRM were stored at room temperature at each designated time point (1, 3, and 5 days); three bottles were randomly selected and analyzed. For the long‐term stability evaluation, three bottles of the candidate CRM were randomly chosen from storage at −18°C and analyzed at intervals of 0, 1, 3, 6, 9, and 12 months. At each time point, three subsamples from the selected bottles were analyzed in triplicate using GC‐FID. The stability study results were assessed by performing analysis of variance (ANOVA) on linear regression data to verify trends of the purity of the GLA‐ME candidate CRM (ISO 2016,2017).

## 3 Results and Discussion

### 3.1 Preparation of the Candidate CRM

GLA is derived from the seed oils of borage ( *B. officinalis* ), blackcurrant ( *R. nigrum* ), and evening primrose ( *O. biennis* ). Among these, EPO serves as the primary source of GLA. EPO is rich in PUFAs, including linoleic acid (60%–65%), GLA (7%–14%), as well as oleic, palmitic, and stearic acids (Li et al. 2018; Hsieh et al. 2024; Shahinfar et al. 2023). EPO is extracted from the seeds of the evening primrose plant ( *O. biennis* ) found in China and Central America. So, EPO was chosen as the raw material source for GLA‐ME in the study. GLA exists in its acid form within EPO. This acid form of GLA in EPO serves as the foundational raw material for further processing or derivatization, such as conversion to GLA‐ME for specific applications. Therefore, the initial step in preparing GLA‐ME involves the methylation of GLA present in EPO. The reaction conditions for methylation of GLA to GLA‐ME were slightly modified based on the reported literature method (Fang et al. 2020). This optimization process included adjusting the ratio of GLA to the methylation reagent, refining the reaction temperature, and determining the optimal reaction time to achieve efficient and effective methylation. These modifications ensured an improved yield and quality of GLA‐ME for further study or application. After 300 g EPO was reacted, the reaction solution separated into two distinct layers. The upper layer appeared as a milky white liquid, while the lower layer consisted of a yellow oily liquid containing GLA‐ME. So, the yellow oily liquid was collected for further purification by MD. MD is a physical separation method, which is usually used for the distillation of separating thermosensible compounds to obtain concentrates of value‐added products, and it is the most economically feasible method of purification (Mahrous and Farag 2021). It plays an irreplaceable role in separating components close to the boiling point. By leveraging differences in boiling points, MD enables efficient separation and purification of complex mixtures, making it widely applicable in industries such as petrochemicals, pharmaceuticals, food, and chemicals (García‐Fajardo et al. 2023; Liu et al. 2024).

**FIGURE 1:**
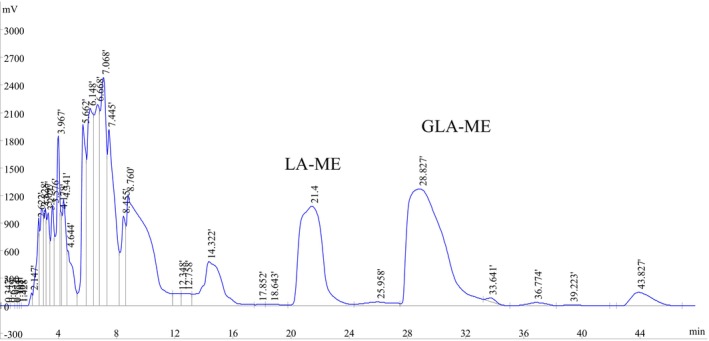
Chromatographic preparation of GLA‐ME on an ZORBAX‐SB‐C18.

Before molecular distillation (MD) purification, key parameters were systematically optimized. For evaporation temperature, tests at 85°C–100°C (fixed conditions: 175 rpm, 30°C condenser, 4 mL min^−1^ feed rate, 1.0 × 10^−3^ mbar) revealed 95°C as optimal, yielding 45% recovery and 58% purity in the light fraction. At 100°C, recovery dropped to 42% due to thermal degradation. Regarding the rotational speed, trials at 150, 175, and 200 rpm indicated that 175 rpm achieved a balance between mass transfer and film stability, with 50% yield and 48% purity. The inner condenser temperature was tested at 35°C, 40°C, and 45°C. The inner condenser temperature of 40°C delivered 55% yield and 53% purity. For the feed rate, tests at 4, 6, and 8 mL min^−1^ demonstrated that 6 mL min^−1^ achieved an ideal balance between separation efficiency and production efficiency, with a yield and purity of 47% and 58%. The vacuum pressure of 1.0 × 10^−3^ mbar was selected based on preliminary trials, as it provided a good vacuum degree to promote distillation without causing excessive energy consumption or equipment requirements. Therefore, the reaction mixture was purified under the optimized conditions: evaporation temperature of 95°C, rotational speed of 175 rpm, inner condenser temperature of 40°C, feed rate of 6 mL min^−1^, and vacuum pressure of 1.0 × 10^−3^ mbar. The light component was collected and then further purified through a secondary MD process under slightly adjusted conditions optimized by the same above process. The resulting light component from the second MD contained over 70% GLA‐ME. To achieve higher purity, this fraction was subsequently purified using optimized Pre HPLC. The chromatogram of the Pre HPLC separation for GLA‐ME is shown in Figure 1. The eluents collected from the preparative separation were concentrated and analyzed to confirm purity. Following Pre HPLC purification, the purity of GLA‐ME exceeded 98% (Figure S1). Finally, the purified eluents were subjected to spin distillation under reduced pressure to remove residual solvents. The resulting GLA‐ME was sealed in brown ampoules, each containing approximately 50 mg, under nitrogen (N_2_) protection to ensure stability and prevent degradation. These ampoules were stored at −18°C as the GLA‐ME candidate CRM for further use in stability studies and analytical applications.

### 3.2 Structure Determination

#### 3.2.1 HRMS Analysis

**FIGURE 2:**
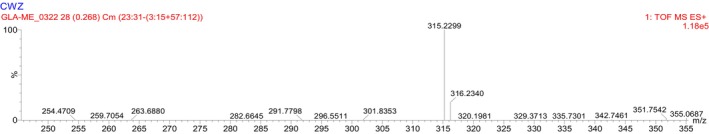
The HRMS spectrum of the GLA‐ME candidate CRM.

HRMS analysis of the GLA‐ME candidate CRM was conducted in positive ion mode. As shown in Figure 2, the measured *m/z* of the candidate CRM was 315.2299, which was consistent with the theoretical value of sodium‐added ion peak [M + Na]^+^ for GLA‐ME, calculated as 315.2300 for the molecular formula C_19_H_32_O_2_Na^+^.

#### 3.2.2 IR Analysis

**FIGURE 3:**
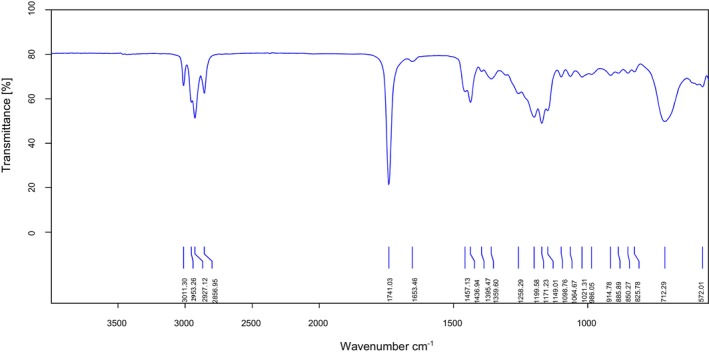
The FT‐IR spectrum of the GLA‐ME candidate CRM.

As can be seen from Figure 3, the IR spectra of the GLA‐ME candidate CRM reveal several characteristic absorption peaks that align with its molecular structure. The absorption peak at 3011 cm^−1^ corresponds to the C‐H stretching vibration in the C=C bond, while the absorption peak at 1436 cm^−1^ is attributed to the C‐C stretching vibration of the C=C bond. The absorption peaks at 2927 and 2856 cm^−1^ represent the C‐H symmetric and asymmetric stretching vibration in alkane CH_2_. The strong absorption peak at 1741 cm^−1^ is indicative of the C=O stretching vibration of a carboxylate ester, and the peak at 1171 cm^−1^ corresponds to the C‐O stretching vibrations in the ester functional group, suggesting the presence of a carboxylate structure in the molecule. Additionally, the absorption peak at 712 cm^−1^ is assigned to the C‐H bending vibration of cis‐HC=CH‐, confirming the presence of an olefinic (cis‐alkene) structure in the molecule. Overall, the IR spectra are consistent with the expected structure of GLA‐ME, confirming the presence of key functional groups such as the ester carbonyl group, alkene bonds, and alkyl chains, which are characteristic of GLA‐ME.

#### 3.2.3 NMR Analysis

**FIGURE 4:**
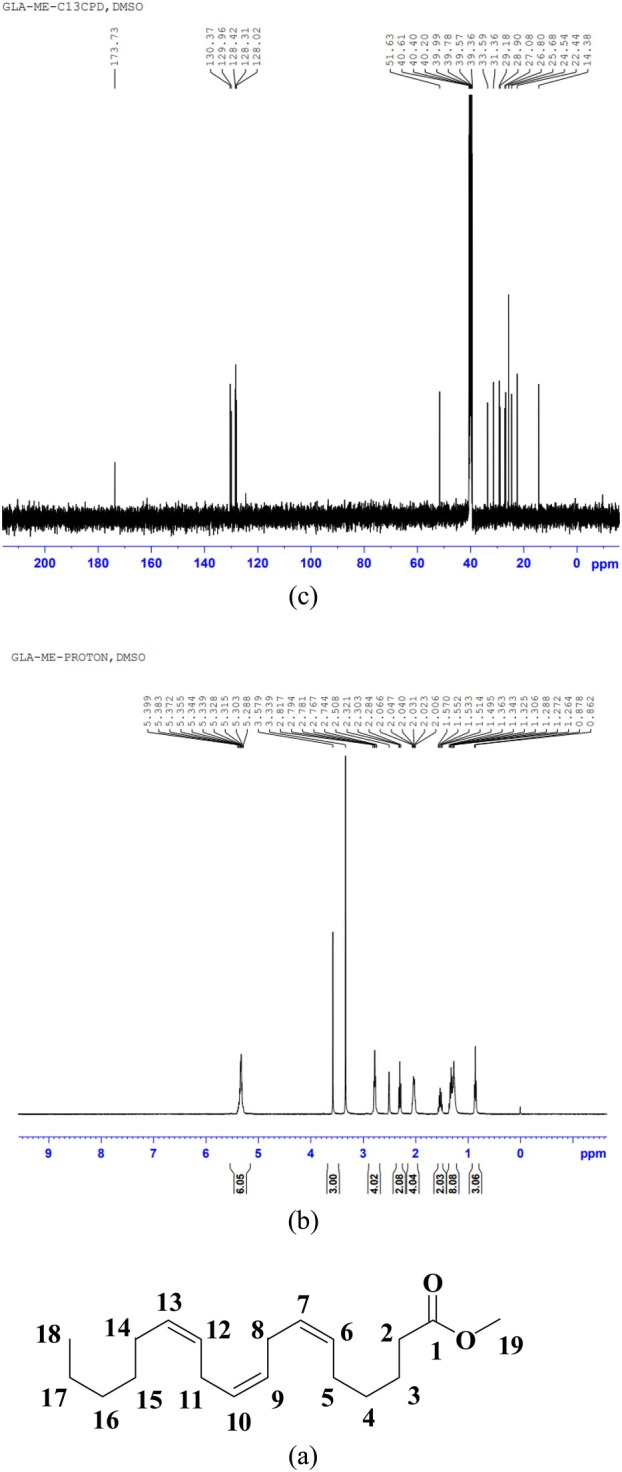
NMR spectra of the GLA‐ME candidate CRM: (a) the structure of GLA‐ME, (b) ^1^H NMR spectra, (c) ^13^C NMR.

The chemical structure and the NMR spectrum of the GLA‐ME candidate CRM are presented in Figure 4a–c. The NMR spectrum results for the GLA‐ME candidate CRM are summarized in Table 1. Based on the analysis, the observed chemical shifts and splitting patterns in the NMR spectrum are consistent with the expected structure of GLA‐ME. The results confirm the presence of characteristic functional groups and molecular framework, including the ester carbonyl group, alkene bonds, and alkyl chains, which align with the theoretical structure of GLA‐ME.

**TABLE 1:** The NMR spectrum of the GLA‐ME candidate CRM (DMSO*‐d*_6_, TMS).

Assignment	*δ*_C_ (100 MHz)	*δ*_H_ (multiplicity, *J* Hz) (400 MHz)
1	173.73	—
2, 3, 4, 5	29.18	2.30 (t, 2H, *J* = 7.2 Hz, CH_2_)
	24.54	1.57–1.51 (m, 2H, CH_2_)
	25.68	1.36–1.26 (m, 2H, CH_2_)
	27.08	2.05–2.01 (m, 2H, CH_2_)
6	129.96	5.40–5.29 (m, 2H)
7	128.31	
8	33.59	2.82–2.74 (m, 2H, CH_2_)
9	128.31	5.40–5.29 (m, 2H)
10	128.42	
11	31.36	2.82–2.74 (m, 2H, CH_2_)
12	128.02	5.40–5.29 (m, 2H)
13	130.37	
14, 15, 16, 17	28.90	1.36–1.26 (m, 6H, 3CH_2_) 2.05–2.01 (m, 2H, CH_2_)
	25.68	
	26.80	
	22.44	
18	14.38	0.86 (t, 3H, *J* = 6.4 Hz, CH_3_)
OCH_3_	51.63	3.58 (s, 3H, OCH_3_)

### 3.3 Optimization and Development of GC‐FID

Several analytical methods are available for the analysis of GLA‐ME, including GC‐FID, GC‐MS, and others (Rydlewski et al. 2021). In this study, a specific GC‐FID method was developed and employed for the value assignment, homogeneity, and stability assessment of the GLA‐ME candidate CRM. To improve the sensitivity, accuracy, and reliability of the measurements, the experimental conditions of GC‐FID were systematically optimized. Key parameters such as the column type, inlet temperature, and temperature programming were carefully investigated and adjusted. Under the optimized chromatographic condition, the main component (GLA‐ME) was baseline separated from other structurally related impurities, as demonstrated in Figure S1. This separation ensured precise quantification and reliable analysis of the GLA‐ME candidate CRM, supporting its characterization as a high‐quality CRM.

After optimizing the chromatographic condition, the performance characteristics of the GC‐FID method, including the linear range, reproducibility, limit of detection (LOD), and limit of quantitation (LOQ), were evaluated to ensure its suitability for purity assessment. The developed method demonstrated an excellent linear response across a concentration range of 0.20–6.00 mg·mL^−1^, as indicated by the regression equation *y* = 2037.50*x*−191.11 with a correlation coefficient (*R* = 0.9982). The mean residual value of 0.02, being negligibly close to zero, further confirms the model's minimal systematic error and robustly validates both the reliability of the calibration curve fitting and the accuracy of the analytical method. The LOD was determined to be 0.08 μg·mL^−1^, established by progressively diluting the standard solution until a S/N of 3:1 was achieved. Similarly, the LOQ was found to be 0.20 μg·mL^−1^, determined at an S/N ratio of 10:1. The reproducibility of the method was assessed through six replicate measurements, yielding a relative standard deviation (RSD) of 0.05% (Table 2). These results confirm the method's precision, sensitivity, and reliability for the accurate quantification and purity assessment of the GLA‐ME candidate CRM. The certified concentrations of the candidate CRM were further optimized by evaluating the purity and impurity profiles at varying concentrations. The results indicated that the purity and impurity levels of the candidate CRM remained consistent and stable at concentrations greater than 1.00 mg·mL^−1^. Based on this finding, a concentration of 2.00 mg·mL^−1^ was selected as the certified concentration for the candidate CRM. This concentration ensures optimal stability and reliability for the characterization and use of the CRM in analytical applications.

**TABLE 2:** Determination results for the GLA‐ME candidate CRM.

Number	GC‐FID (%)	Moisture (%)	Volatile impurities (%)	Nonvolatile impurities (%)	Determination results	
					MB (%)	*q*NMR (%)
1	99.28	0.245	< 0.01	< 0.01	99.04	98.96
2	99.24	0.258	< 0.01	< 0.01	99.02	99.10
3	99.23	0.325	< 0.01	< 0.01	98.96	99.06
4	99.24	0.333	< 0.01	< 0.01	98.95	99.00
5	99.26	0.273	< 0.01	< 0.01	99.01	98.96
6	99.26	0.303	< 0.01	< 0.01	98.98	99.04
Mean	99.25	0.290	—	—	98.96	99.02
SD	0.02	0.036	—	—	0.04	0.06
$C_{\text{calculate}}$	0.8287					
$C_{\left(0.05,2,6\right)}$	0.8534					
Conclusion	$C_{\text{calculate}}$ < $C_{0.5,6}$, the two methods are equiprecise					
$t_{\text{calculate}}$	0.45					
$t_{\left(0.05,10\right)}$	2.23					
Conclusion	$F_{\text{calculate}}$ < $F_{0.5\left(5,5\right)}$, the means are coincident					

### 3.4 Homogeneity Test

Table S1 summarizes the homogeneity assessment results for the GLA‐ME candidate CRM. According to ISO Guide 35:2017 (ISO 2017), the data were evaluated using ANOVA with an *F*‐test. The calculated *F*‐value was 1.86, which is smaller than the critical value. This indicates that there were no significant differences in the homogeneity of the GLA‐ME candidate CRM during the experimental period, as further detailed in Table S2. The results confirm that the candidate CRM exhibits sufficient homogeneity, meeting the requirements for use as a CRM.

### 3.5 Stability Test

In this study, the stability (a vital CRM attribute) of the GLA‐ME candidate CRM was evaluated under both long‐term storage conditions (ambient temperature) and short‐term transportation conditions (room temperature). The purity of the GLA‐ME candidate CRM was monitored over time to assess any fluctuations. At predetermined time points and under different temperature conditions, three bottles were sampled, and three subsamples from each bottle were analyzed in triplicate using the developed GC‐FID method. The results are detailed in Tables S3 and S4. The slope (b1) and the standard uncertainty of the slope s(b1) were evaluated through linear regression. The absolute b1 value was below the product of s(b1) and t(0.95, *n*‐2). This indicates that there were no significant trends in the purity of the GLA‐ME candidate CRM over time. The results demonstrate that the purity of the GLA‐ME candidate CRM was maintained for at least 12 months under −18°C setting and for 5 days at room temperature without any special precautions.

### 3.6 MB Approach

The results obtained by MB are summarized in Table 2. The content of the main component was 99.25% ± 0.02% using GC‐FID. The moisture content determined by Karl‐Fischer titration was found to be 0.290% ± 0.036%. Additionally, the volatile impurities measured by head‐space GC‐FID and the nonvolatile impurities measured using ICP‐MS were both less than 0.01%, indicating that these impurities are negligible for the GLA‐ME candidate CRM. The average purity of the GLA‐ME candidate CRM determined by MB is 98.96%, calculated according to Equation (1).

### 3.7 qNMR Method

**FIGURE 5:**
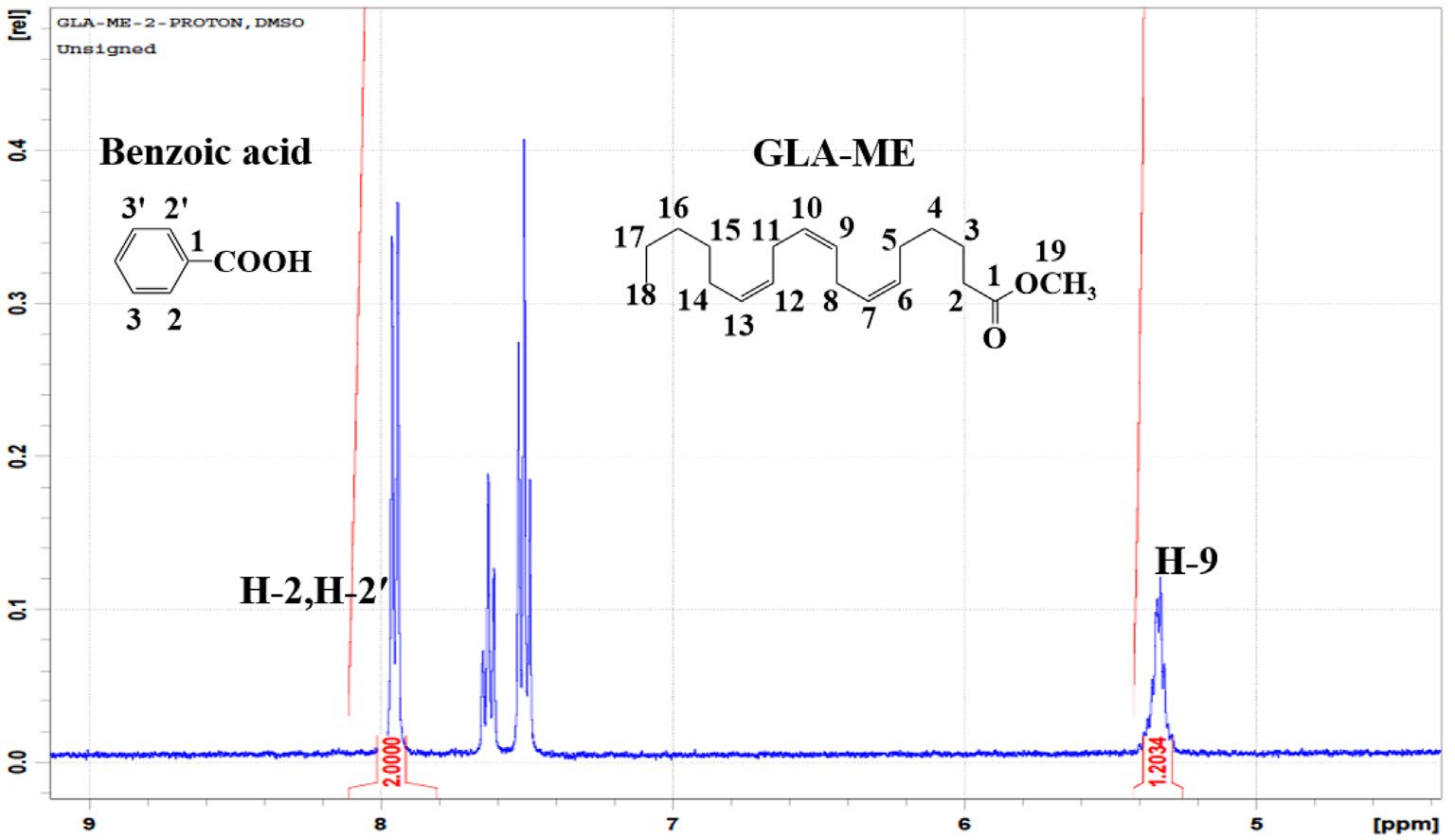
^1^H NMR spectrum of the GLA‐ME candidate CRM and benzoic acid in DMSO‐d6.

The results obtained from the MB approach were further verified using *q*NMR. *q*NMR is a precise, non‐destructive analytical tool that offers the advantages of high precision, reduced solvent use, and simple sample preparation. Unlike GC‐FID or HPLC, it does not require a pure target analyte as a reference material for calibration purposes. In *q*NMR, for a given number of analytes, the signal region is directly related to the number of nuclei present. There are two types of *q*NMR: relative *q*NMR, which measures the ratio of integral values of different components in a mixture, and absolute *q*NMR, which measures the actual quantity and purity of the analytes in the mixture (Nayak et al. 2024). In the study, an absolute *q*NMR with an IS was developed to determine the purity of the GLA‐ME candidate CRM. Benzoic acid CRM (GBW 06117) was selected as IS because its ^1^H NMR signal does not overlap with those of the GLA‐ME candidate CRM. As shown in Figure 5, the peaks corresponding to the protons on the CH=CH groups of GLA‐ME (*δ* = 5.40–5.29) and the aromatic proton of benzoic acid (*δ* = 8.05) were well‐separated from other peaks, ensuring accurate integration of their peak areas. The purity of the GLA‐ME candidate CRM was determined by comparing the signal integration at *δ* = 5.40–5.29 (CH=CH) of the GLA‐ME candidate CRM and the signal integration at δ 8.05 (CH = CH) of the IS. Six specimens of the GLA‐ME candidate CRM were selected and analyzed. The mean purity value (*n* = 6) of the GLA‐ME candidate CRM determined by *q*NMR is listed in Table 2.

### 3.8 Purity Certification

The comparison between the results from MB and *q*NMR was statistically evaluated using the William Gemmell Cochran test and the *t*‐test. As shown in Table 2, $C_{\text{calculate}}$ = 0.8287 is lower than $C_{\left(0.05,2,6\right)}=0.8534$, and $t_{\text{calculate}}$ = 0.45 is lower than $t_{\left(0.05,10\right)}$=2.23. This indicated that there is no obvious difference between the two methods. Therefore, the defined value for the purity of the GLA‐ME candidate CRM was calculated as the mean of the results from both methods (ISO 2017).

(4) $$P=\frac{P_{\mathrm{MB}}+P_{\text{qNMR}}}{2}=\frac{98.86\%+99.02\%}{2}\approx 98.99\%$$

### 3.9 Uncertainty Assessment

Following ISO Guide 35, the relative uncertainty of the certified value ($u_{\mathrm{CRM}-\mathrm{rel}}$) can be evaluated according to Equation (5) by combining $u_{\mathrm{char}-\mathrm{rel}}$, $u_{\mathrm{bb}-\mathrm{rel}}$, $u_{\mathrm{lst}-\mathrm{rel}}$ (the relative uncertainty of long‐term stability) and $u_{\mathrm{sst}-\mathrm{rel}}$ (the relative uncertainty of short‐term stability) (ISO 2016).

(5) $$u_{\mathrm{CRM}-\mathrm{rel}}=\sqrt{u_{\mathrm{char}-\mathrm{rel}}^{2}+u_{\mathrm{bb}-\mathrm{rel}}^{2}+u_{\mathrm{lst}-\mathrm{rel}}^{2}+u_{\mathrm{sst}-\mathrm{rel}}^{2}}$$

#### 3.9.1 Measurement Uncertainty in MB

The combined uncertainty $u_{\mathrm{MB}-\mathrm{rel}}\left(P\right)$ of the MB can be determined according to Equation (6) (ISO 2008):

(6) $$u_{\mathrm{MB}-\mathrm{rel}}\left(P\right)=\sqrt{{\left[u_{\mathrm{rel}}\left(P_{0}\right)\right]}^{2}+\frac{{\left[u\left(X_{V}\right)\right]}^{2}+{\left[u\left(X_{W}\right)\right]}^{2}+{\left[u\left(X_{\mathrm{NV}}\right)\right]}^{2}}{{\left(1-X_{V}-X_{W}-X_{\mathrm{NV}}\right)}^{2}}}$$

where *P*_0_ and $u_{\mathrm{rel}}\left(P_{0}\right)$ separately denote the main component's content and relative uncertainty determined based on GC‐FID; and *X*_*W*_, *u*(*X*_*W*_), *X*_*V*_, *u*(*X*_*V*_), and *X*_*NV*_, *u*(*X*_*NV*_) represent the contents and uncertainties, severally for moisture, volatile impurities, and nonvolatile impurities. Among them, the $u_{\mathrm{rel}}\left(P_{0}\right)$ parameter, which comprises *u*_*rel*_,_1_, *u*_*rel*,2_, *u*_*rel*_,_3_, and *u*_*rel*,4_, can be formulated by Equation (7):

(7) $$u_{\mathrm{rel}}\left(P_{0}\right)=\sqrt{{u_{\mathrm{rel},1}}^{2}+{u_{\mathrm{rel},2}}^{2}+{u_{\mathrm{rel},3}}^{2}+{u_{\mathrm{rel},4}}^{2}}$$

In Equation (7), $u_{\mathrm{rel},1}$ was calculated as the following:

(8) $$u_{\mathrm{rel},1}=\frac{\mathrm{RSD}}{\sqrt{n}}=\frac{0.02\%}{\sqrt{6}}=0.01\%.$$

Here, *n* is the times of repeated measurements, 0.02% Is the relative SD (RSD) of main component measurements by the GC‐FID method (shown in Table 1).

$u_{\mathrm{rel},2}$ can be expressed as Equation (7) (Station Administration for Market Regulation 2021; Chen, Jin, Zhang, et al. 2022):

(9) $$u_{\mathrm{rel},2}=\frac{u_{2}}{P_{0}}=\frac{1}{P_{0}}\sqrt{\sum_{i=1}^{n}{\left[x_{i}∙\frac{\left|\frac{f_{i}}{f_{0}}-1\right|}{\frac{f_{i}}{f_{0}}}\right]}^{2}}$$

According to the results of GC–MS analysis, three main impurities were identified in the GLA‐ME candidate CRM, with molecular formulas C_19_H_34_O_2_ and C_21_H_34_O_2_, respectively. The three main impurity content in the GLA‐ME purity CRM was determined to be 0.12%, 0.39%, and 0.06%, respectively. Additionally, other unidentified impurities accounted for 0.27% of the total composition, as detailed in Table S5. Since the molecular formulas of these other impurities could not be detected by GC‐MS, the impurity with the largest structural difference from the principal component, C_21_H_34_O_2_, The unidentified impurities were conservatively assigned a value of 0.27% for this calculation. According to Equation (9), the relative uncertainty associated with these impurities $u_{\mathrm{rel},2}$ was calculated to be 0.005%.

*u*_*rel*_,_3_ was evaluated with the linear range of the developed GC‐FID method. The certified concentration of GLA‐ME candidate CRM was 2.00 mg∙mL^−1^, which was within the scope of the linear range (0.20–6.00 mg∙mL^−1^) of the GC‐FID method. Given this, the influence of *u*_*rel*_,_3_ could be negligible. Consequently, it could be safely ignored in subsequent analyses.

*u*_*rel*_,_4_ originated from the sensitivity of GC‐FID was estimated based on its LOD. The equation for *u*_*rel*,4_ was determined by Equation (10) as follows:

(10) $$u_{\mathrm{rel},4}=\mathrm{LOD}/c=0.08\;\mathrm{\mu g}∙{\mathrm{mL}}^{-1}/2.00\;\mathrm{mg}∙{\mathrm{mL}}^{-1}=0.004\%$$

Here, *c* is the certified concentration of GLA‐ME candidate CRM.

Therefore, the main component uncertainty originated from the GC‐FID method for the GLA‐ME candidate CRM was 0.02% calculated by Equation (7) as follows: $u_{\mathrm{rel}}\left(P_{0}\right)=\sqrt{{\left(0.01\%\right)}^{2}+{\left(0.005\%\right)}^{2}+{\left(0.004\%\right)}^{2}}=0.02\%.$

The uncertainty caused by the moisture content $u\left(X_{W}\right)$ was calculated by Equation (11) (Chen, Jin, Fang, et al. 2022).

(11) $$u\left(X_{W}\right)=X_{W}\sqrt{u_{\mathrm{rel},1}^{2}+{\left[\frac{u\left(m\right)}{m}\right]}^{2}+{\left[\frac{u\left(W\right)}{W}\right]}^{2}+{\left[\frac{u\left(f\right)}{f}\right]}^{2}}$$

Here, $X_{W}$ means the moisture content of the GLA‐ME candidate CRM, $u_{\mathrm{rel},1}$ means the SD for moisture measurement; $u\left(m\right)$ and $u\left(W\right)$ denote the uncertainties for the weighed candidate CRM mass and the moisture mass in the candidate CRM, respectively; *m* and *W* represent the weighed candidate CRM mass and moisture mass in the candidate CRM, respectively; The value of f was determined using the correction factor of moisture and temperature, and the $u\left(f\right)$ was standard uncertainty of f.

*u*_*rel*_,_1_ was estimated by the RSD of the moisture measurements calculated as

(12) $$\;u_{\mathrm{rel},1}=\frac{\mathrm{RSD}}{\sqrt{n}}=\frac{12.53\%}{\sqrt{6}}=5.11\%$$

(RSD = 12.53%, *n* indicates the times of the moisture measurement).

$\frac{u\left(m\right)}{m}$ denote the relative uncertainty of the weighed candidate CRM mass. Approximately 40 mg of the candidate CRM was used to determine the weighed mass (m) of the moisture. Here, m is 40 mg. The uncertainty in the mass measurement $u\left(m\right)$ is 0.01 mg, which was obtained by the precision of the used analytical balance (*d* = 0.01 mg). Therefore, $\frac{u\left(m\right)}{m}$ was calculated as follows:

(13) $$\frac{u\left(m\right)}{m}=\frac{0.01\;\mathrm{mg}}{40\;\mathrm{mg}*\sqrt{3}}\times 100\%=0.015\%.$$

$\frac{u\left(W\right)}{W}$ represents the relative uncertainty of the moisture mass in the candidate CRM. The mass of moisture W was calculated as the product of the weighed candidate CRM mass (40 mg) and the moisture content (0.290%) in the candidate CRM. So, W = 40 mg × 0.290% = 0.116 mg = 116 μg. The uncertainty in the moisture measurement $u\left(W\right)$ was 10 μg, which was the resolution of the moisture meter. Therefore, $\frac{u\left(W\right)}{W}$ was calculated as:

(14) $$\frac{u\left(W\right)}{W}=\frac{10\;\mathrm{\mu g}}{116\;\mathrm{\mu g}}\times 100\%=0.086\%$$

$\frac{u\left(f\right)}{f}$ indicates the relative uncertainty caused by the correction coefficient of the moisture meter. Since the moisture meter has been validated by the moisture CRM and the experimental results of the moisture CRM are consistent with the certified values, the relative uncertainty due to the correction factor of the moisture meter can be neglected. So, $u\left(X_{W}\right)$ calculated from Equation (11) was 0.015%.

The nonvolatile and volatile impurities of the candidate CRM were significantly less than 0.01%. Therefore, their corresponding uncertainties in the MB was negligible. Consequently, the combined uncertainty $u_{\mathrm{MB}-\mathrm{rel}}\left(P\right)$ of MB calculated from Equation (6) was 0.03%.

#### 3.9.2 Measurement Uncertainty of qNMR

The combined relative uncertainty $u_{\text{qNMR}-\mathrm{rel}}$ raised from *q*NMR can be calculated according to Equation (15) (ISO 2008).

(15) uqNMR−rel=uPxPx=uIxIstdIxIstd2+uMxMx2+uMstdMstd2+umstdmstd2+umxmx2+uPstdkstd2

Here, uIxIstdIxIstd indicates the area ratio of quantitative peak; M and m indicates molar mass and the weight mass of the compound, respectively. $u\left(M\right)$ and u(m) indicates the uncertainty of the foregoing two. $u\left(P_{\mathrm{std}}\right)$ indicates the uncertainty of IS, $k_{\mathrm{std}}$ indicates the coverage factor of the uncertainty of IS. The subscripts x and std indicates the GLA‐ME candidate CRM and the IS, respectively.

uIxIstdIxIstd can be calculated as the following:

(16) uIxIstdIxIstd=RSDn=0.04%6=0.02%,

where *n* is the number of repeated measurements by *q*NMR. Given that *n* = 6 and the RSD = 0.04%, uIxIstdIxIstd was calculated as

(17) $$\frac{\mathrm{RSD}}{\sqrt{n}}=\frac{0.04\%}{\sqrt{6}}=0.02\%$$

$\frac{u\left(M_{x}\right)}{M_{x}}\;\text{and}\;\frac{u\left(M_{\mathrm{std}}\right)}{M_{\mathrm{std}}}$ were 0.003% and 0.003%, which indicate the relative uncertainty of molar mass associated with the GLA‐ME candidate CRM and IS, respectively (Station Administration for Market Regulation 2021). $\frac{u\left(m_{x}\right)}{m_{x}}$ and $\frac{u\left(m_{\mathrm{std}}\right)}{m_{\mathrm{std}}}$ indicates the relative uncertainty occurred from the weight mass associated with the GLA‐ME candidate CRM and IS, respectively. Here, they were calculated as follows:

(18) $$\frac{u\left(m_{x}\right)}{m_{x}}=\frac{0.01\;\mathrm{mg}}{5.00\mathrm{mg}*\sqrt{3}}=0.12\%$$

(19) $$\frac{u\left(m_{\mathrm{std}}\right)}{m_{\mathrm{std}}}=\frac{0.01\;\mathrm{mg}}{5.00\mathrm{mg}*\sqrt{3}}=0.12\%$$

Here, 0.01 mg was the sensitivity of the used analytical balance, 5.00 and 5.00 mg were the weight mass of IS and the GLA‐ME candidate CRM, respectively. The relative uncertainty associated with the IS ($\frac{u\left(P_{\mathrm{std}}\right)}{k_{\mathrm{std}}}$) was 0.01%, which can be obtained from the certification of IS. Based on the above results, the relative combined uncertainty ($u_{\text{qNMR}-\mathrm{rel}}$) was calculated according to the Equation (15) as follows:

$$u_{\text{qNMR}-\mathrm{rel}}=\sqrt{{\left(0.02\%\right)}^{2}+{\left(0.003\%\right)}^{2}+{\left(0.003\%\right)}^{2}+{\left(0.01\%\right)}^{2}+{\left(0.12\%\right)}^{2}+{\left(0.12\%\right)}^{2}}=0.18\%.$$

The results of relative uncertainties for the GLA‐ME candidate CRM are shown in Table 2.

#### 3.9.3 Uncertainty of Homogeneity

The uncertainty of heterogeneity ($u_{\mathrm{bb}})$ consist of the homogeneity within the bottles (${\mathrm{MS}}_{\text{within}}$) and the homogeneity between the bottles (${\mathrm{MS}}_{\text{among}}$). In this study, ${\mathrm{MS}}_{\text{among}}$ > ${\mathrm{MS}}_{\text{within}}$, so $u_{\mathrm{bb}}$ was calculated according to Equation (20) (Shao et al. 2022; Guo et al. 2024):

(20) $$u_{\mathrm{bb}=}\sqrt{\frac{{\mathrm{MS}}_{\text{among}}-{\mathrm{MS}}_{\text{within}}}{n}}=\sqrt{\frac{0.001168-0.000619}{3}}=0.02\%$$

The relative uncertainty $u_{\mathrm{bb}-\mathrm{rel}}$ was 0.03% calculated according to $u_{\mathrm{bb}-\mathrm{rel}}=\frac{u_{\mathrm{bb}}}{P}$.

#### 3.9.4 Uncertainty of Stability

The uncertainty of stability $u_{s}$, which consists of the uncertainties from short‐term stability ($u_{\mathrm{sts}}$) and long‐term stability ($u_{\mathrm{lst}}$), is formulated as shown below: $u_{s}$ = *s*(*b*_1_) *t* (21). Here, s(*b*_1_) is a parameter related to the stability characteristics, and t is a variable, often related to time. For the short‐term stability, $u_{\mathrm{sts}}=0.016593\%\times 5=0.09\%.$ For the short‐term stability, $u_{\mathrm{lts}}=0.002733\%\times 12=0.04\%.$ The computational formulas for relative uncertainties of short‐term stability $u_{\mathrm{sts}-\mathrm{rel}}$ and long‐term stability $u_{\mathrm{lst}-\mathrm{rel}}$ were: $u_{\mathrm{sts}-\mathrm{rel}}=\frac{u_{\mathrm{sts}}}{P}=\frac{0.09\%}{98.99\%}=0.10\%\text{and}\;u_{\mathrm{lst}-\mathrm{rel}}=\frac{u_{\mathrm{lst}}}{P}=\frac{0.04\%}{98.99\%}=0.05\%.$

#### 3.9.5 Combined and Expanded Uncertainties

In the light of ISO guide 35 principles (ISO 2016), the combined relative uncertainty $u_{\mathrm{CRM}-\mathrm{rel}}$ is derivable as the integration of the relative uncertainties from the stability and homogeneity tests, as well as characterization, and can be obtained according to Equation (5): $u_{\mathrm{CRM}-\mathrm{rel}}=\sqrt{{\left(0.11\%\right)}^{2}+{\left(0.03\%\right)}^{2}+{\left(0.05\%\right)}^{2}+{\left(0.10\%\right)}^{2}}=0.17\%$. Expanded relative uncertainty $U_{\mathrm{CRM}-\mathrm{rel}}$ was calculated as follows: $U_{\mathrm{CRM}-\mathrm{rel}}=u_{\mathrm{CRM}-\mathrm{rel}}\times k$ (21) (*k =* 2, representing coverage factor). Expanded uncertainty $U_{\mathrm{CRM}}$ was calculated as follows: $U_{\mathrm{CRM}}=u_{\mathrm{CRM}-\mathrm{rel}}\times P$. Table 3 details the results.

**TABLE 3:** The uncertainties of the GLA‐ME candidate CRM.

Uncertainties	Results (%)
$u_{\mathrm{bb}-\mathrm{rel}}$	0.05
$u_{\mathrm{lts}-\mathrm{rel}}$	0.05
$u_{\mathrm{sts}-\mathrm{rel}}$	0.10
$u_{\mathrm{rel}}\left(P_{\text{qNMR}}\right)$	0.18
$u_{\mathrm{rel}}\left(P_{\mathrm{MB}}\right)$	0.03
$u_{\mathrm{char}-\mathrm{rel}}$	0.11
$u_{\mathrm{CRM}-\mathrm{rel}}$	0.17
$U_{\mathrm{CRM}-\mathrm{rel}}$	0.34
$U_{\mathrm{CRM}}$	0.4

## 4 Conclusion

The GLA‐ME CRM has been approved by the State Administration for Market Regulation of the People's Republic of China. This pure CRM was assigned the number GBW (E) 091214. The certified purity of GLA‐ME CRM was 98.9% ± 0.4% determined by MB and *q*NMR. Karl–Fischer titration was used for the determination of water in GLA‐ME, while ICP‐MS and head‐space GC‐FID were used for the determination of the nonvolatile and volatile impurities, respectively. The GLA‐ME CRM was found to be homogeneous and stable at room temperature for 5 days and at −18°C for at least 12 months. The CRM stability will be continuously monitored in the future, and the validity date will be extended based on the test results. Given that this GLA‐ME purity CRM features high purity, along with stability and homogeneity, we are confident that it will play a crucial role in standardizing the measurements of GLA in foods.

## Author Contributions

**Weizhu Chen:** conceptualization (equal), funding acquisition (equal), methodology (equal), writing – original draft (equal), writing – review and editing (equal). **Wenhui Jin:** formal analysis (equal). **Hua Fang:** data curation, Validation (equal). **Hui Chen:** investigation (equal), resources (equal). **Xiaoyuan Huang:** data curation (equal), formal analysis (equal), validation (equal). **Yanrou Jie:** data curation (equal). **Zhuan Hong:** project administration (equal), resources (equal). **Yiping Zhang:** funding acquisition (equal), writing – review and editing (equal).

## Conflicts of Interest

The authors declare no conflicts of interest.

## Supporting information


**Figure S1.** The GC‐FID chromatogram of GLA‐ME candidate CRM: (a) Full image; (b) enlarged image.

**FIGURE S2.** The process for developing GLA‐ME CRM.

**Table S1.** Homogeneity test results of the GLA‐ME candidate CRM (%).

**Table S2.** ANOVA analysis of homogeneity test results.

**Table S3.** Long‐term stability results of the GLA‐ME candidate CRM.

**Table S4.** Short‐term stability results of the GLA‐ME candidate CRM.

**Table S5.** The molecular formula and the content of the components in the GLA‐ME candidate CRM.

## Data Availability

Research data are not shared.
