# Supplementary material for: Development of a New Purity Certified Reference Material of Gamma Linolenic Acid Methyl Ester
Source: Food Sci Nutr. 2025 Jun 5;13(6):e70354. doi: 10.1002/fsn3.70354 (PMC12138581; doi:10.1002/fsn3.70354)
Supplement: Supplementary file 1 — Figure S1. The GC‐FID chromatogram of GLA‐ME candidate CRM: (a) Full image; (b) enlarged image. [file FSN3-13-e70354-s007.docx]

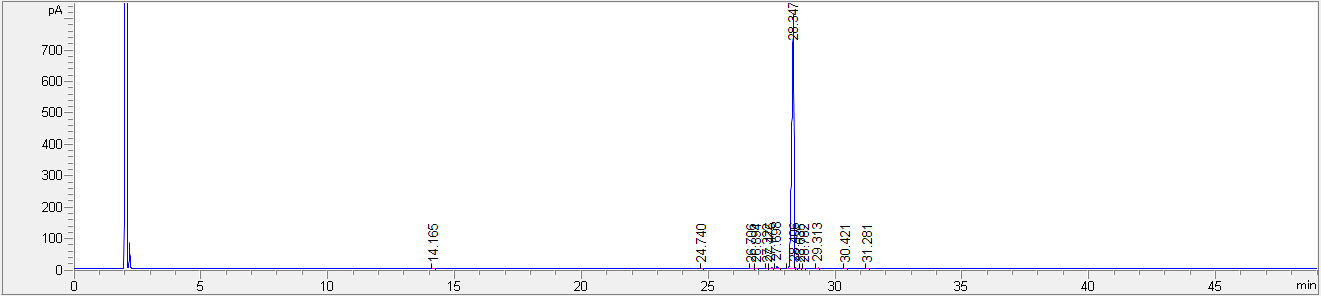
 (a)

LA-ME

GLA-ME


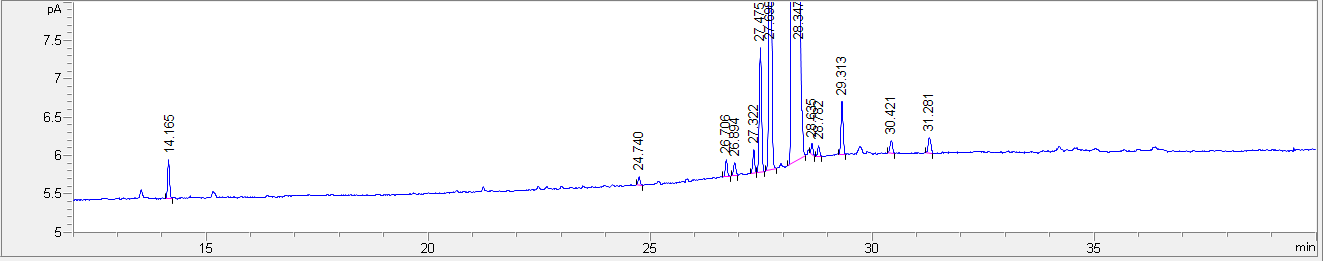


LA-ME

GLA-ME

(b)

Figure S1 The GC-FID chromatogram of GLA-ME candidate CRM:(a) Full image; (b) enlarged image
