# Supplementary figures and images for: Development of a New Purity Certified Reference Material of Gamma Linolenic Acid Methyl Ester
Source: Food Sci Nutr. 2025 Jun 5;13(6):e70354. doi: 10.1002/fsn3.70354 (PMC12138581; doi:10.1002/fsn3.70354)

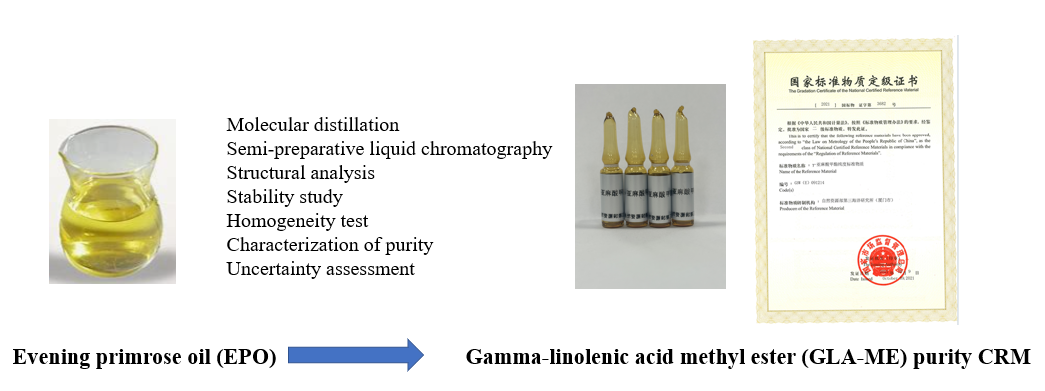


Figure S2 the process for developing GLA-ME CRM

Supplement: Supplementary file 2 — FIGURE S2. The process for developing GLA‐ME CRM. [file FSN3-13-e70354-s003.docx]
