# Supplementary material for: Development of a New Purity Certified Reference Material of Gamma Linolenic Acid Methyl Ester
Source: Food Sci Nutr. 2025 Jun 5;13(6):e70354. doi: 10.1002/fsn3.70354 (PMC12138581; doi:10.1002/fsn3.70354)
Supplement: Supplementary file 3 — Table S1. Homogeneity test results of the GLA‐ME candidate CRM (%). [file FSN3-13-e70354-s004.docx]

Table S1 Homogeneity test results of the GLA-ME candidate CRM (%)

| ***Number*** | **1** | **2** | **3** | **Means** |
| --- | --- | --- | --- | --- |
| i=1 | 99.17 | 99.20 | 99.21 | 99.19 |
| i=2 | 99.22 | 99.21 | 99.18 | 99.20 |
| i=3 | 99.26 | 99.21 | 99.24 | 99.24 |
| i=4 | 99.17 | 99.20 | 99.20 | 99.19 |
| i=5 | 99.20 | 99.20 | 99.21 | 99.20 |
| i=6 | 99.21 | 99.18 | 99.23 | 99.20 |
| i=7 | 99.19 | 99.18 | 99.21 | 99.19 |
| i=8 | 99.24 | 99.22 | 99.22 | 99.22 |
| i=9 | 99.20 | 99.15 | 99.23 | 99.19 |
| i=10 | 99.24 | 99.18 | 99.22 | 99.21 |
| i=11 | 99.10 | 99.14 | 99.22 | 99.15 |
| i=12 | 99.17 | 99.18 | 99.16 | 99.17 |
| i=13 | 99.20 | 99.21 | 99.22 | 99.21 |
| i=14 | 99.19 | 99.19 | 99.24 | 99.21 |
| i=15 | 99.21 | 99.20 | 99.20 | 99.20 |
| Overall mean  meanmeanmean 99.85 | 99.20 |  |  |  |
| Standard deviation dedeviation 0.0086 | 0.03 |  |  |  |
