# Supplementary material for: Development of a New Purity Certified Reference Material of Gamma Linolenic Acid Methyl Ester
Source: Food Sci Nutr. 2025 Jun 5;13(6):e70354. doi: 10.1002/fsn3.70354 (PMC12138581; doi:10.1002/fsn3.70354)
Supplement: Supplementary file 4 — Table S2. ANOVA analysis of homogeneity test results. [file FSN3-13-e70354-s006.docx]

Table S2 ANOVA analysis of homogeneity test results

| **Parameters** | **Values** |
| --- | --- |
| Mean square between bottles/$s_{1}^{2}$ | 0.001168 |
| Mean square within bottles/$s_{2}^{2}$ | 0.000629 |
| F/ ($s_{1}^{2}$/ $s_{2}^{2}$) | 1.86 |
| F_0.05_ (14, 30) | 2.04 |
| Conclusion | F < F_0.05_ (14, 30) |
