# Supplementary material for: Development of a New Purity Certified Reference Material of Gamma Linolenic Acid Methyl Ester
Source: Food Sci Nutr. 2025 Jun 5;13(6):e70354. doi: 10.1002/fsn3.70354 (PMC12138581; doi:10.1002/fsn3.70354)
Supplement: Supplementary file 5 — Table S3. Long‐term stability results of the GLA‐ME candidate CRM. [file FSN3-13-e70354-s001.docx]

Table S3 Long-term stability results of the GLA-ME candidate CRM

| **Time (month)** | **Purity**  **(%)** | **Averge**  **(%)** | $\boldsymbol{b}_{\boldsymbol{1}}$ | $\boldsymbol{b}_{\boldsymbol{0}}$ | $\boldsymbol{s}$ | **s(b_1_)** | **t_0.95,n–2_** | **Conclusion** |
| --- | --- | --- | --- | --- | --- | --- | --- | --- |
| 0 | 99.23 | 99.24 | -0.002752 | 99.26 | 0.028769 | 0.002733 | 2.776 | \|b1\|<t⋅s(b1),stable |
| 1 | 99.27 |  |  |  |  |  |  |  |
| 3 | 99.28 |  |  |  |  |  |  |  |
| 6 | 99.22 |  |  |  |  |  |  |  |
| 9 | 99.26 |  |  |  |  |  |  |  |
| 12 | 99.21 |  |  |  |  |  |  |  |
