# Supplementary material for: Development of a New Purity Certified Reference Material of Gamma Linolenic Acid Methyl Ester
Source: Food Sci Nutr. 2025 Jun 5;13(6):e70354. doi: 10.1002/fsn3.70354 (PMC12138581; doi:10.1002/fsn3.70354)
Supplement: Supplementary file 6 — Table S4. Short‐term stability results of the GLA‐ME candidate CRM. [file FSN3-13-e70354-s005.docx]

Table S4 Short-term stability results of the GLA-ME candidate CRM

| **Time (day)** | **Purity**  **(%)** | **Average**  **(%)** | $\boldsymbol{b}_{\boldsymbol{1}}$ | $\boldsymbol{b}_{\boldsymbol{0}}$ | $\boldsymbol{s}$ | **s(b_1_)** | **t_0.95,n–2_** | **Conclusion** |
| --- | --- | --- | --- | --- | --- | --- | --- | --- |
| 0 | 99.23 | 99.19 | -0.001678 | 99.22 | 0.063726 | 0.028455 | 4.303 | \|b_1_\|<t⋅s(b_1_), stable |
| 1 | 99.24 |  |  |  |  |  |  |  |
| 3 | 99.10 |  |  |  |  |  |  |  |
| 5 | 99.18 |  |  |  |  |  |  |  |
