# Supplementary material for: Development of a New Purity Certified Reference Material of Gamma Linolenic Acid Methyl Ester
Source: Food Sci Nutr. 2025 Jun 5;13(6):e70354. doi: 10.1002/fsn3.70354 (PMC12138581; doi:10.1002/fsn3.70354)
Supplement: Supplementary file 7 — Table S5. The molecular formula and the content of the components in the GLA‐ME candidate CRM. [file FSN3-13-e70354-s002.docx]

| **component** | **Molecular formula** | **Content（%）** |
| --- | --- | --- |
| Main component (squalene) | C_19_H_32_O_2_ | 99.16 |
| Impurity 1 | C_19_H_34_O_2_ (R_t_=27.471 min) | 0.12 |
| Impurity 2 | C_19_H_34_O_2_ (R_t_=27.695 min) | 0.39 |
| Impurity 3 | C_21_H_34_O_2_ (R_t_=29.311 min) | 0.06 |
| other impurities | undetermined | 0.27 |

Table S5 The molecular formula and the content of the components in the GLA-ME candidate CRM
